# The role of comprehensive analysis with circulating tumor DNA in advanced non‐small cell lung cancer patients considered for osimertinib treatment

**DOI:** 10.1002/cam4.3929

**Published:** 2021-05-12

**Authors:** Naoko Sueoka‐Aragane, Chiho Nakashima, Hironori Yoshida, Naohisa Matsumoto, Kentaro Iwanaga, Noriyuki Ebi, Akihiro Nishiyama, Kazuhiro Yatera, Shoichi Kuyama, Minoru Fukuda, Sunao Ushijima, Hitomi Umeguchi, Daijiro Harada, Kosuke Kashiwabara, Takayuki Suetsugu, Nobukazu Fujimoto, Fumihiro Tanaka, Hidetaka Uramoto, Chiharu Yoshii, Katsumi Nakatomi, Genju Koh, Nobuhiko Seki, Keisuke Aoe, Kaname Nosaki, Koji Inoue, Ayako Takamori, Atsushi Kawaguchi

**Affiliations:** ^1^ Division of Hematology, Respiratory Medicine and Oncology Department of Internal Medicine Faculty of Medicine Saga University Saga Japan; ^2^ Department of Respiratory Medicine Graduate School of Medicine Kyoto University Kyoto Japan; ^3^ Department of Respiratory Medicine Juntendo University Graduate School of Medicine Tokyo Japan; ^4^ Department of Respiratory medicine Saga‐Ken Medical Centre Koseikan Saga Japan; ^5^ Department of Respiratory Medicine Iizuka Hospital Fukuoka Japan; ^6^ Division of Medical Oncology Cancer Research Institute Kanazawa University Ishikawa Japan; ^7^ Department of Respiratory Medicine University of Occupational and Environmental Health Fukuoka Japan; ^8^ Department of Respiratory Medicine National Hospital Organization Iwakuni Clinical Center Yamaguchi Japan; ^9^ Department of Respiratory Medicine Nagasaki University Hospital Nagasaki Japan; ^10^ Department of Respiratory Medicine Kumamoto Chuo Hospital Kumamoto Japan; ^11^ Department of respiratory medicine Karatsu Red Cross Hospital Saga Japan; ^12^ Department of Thoracic Oncology National Hospital Organization Shikoku Cancer Center Ehime Japan; ^13^ Department of Respiratory Medicine Kumamoto Regional Medical Center Kumamoto Japan; ^14^ Department of Respiratory Medicine Sendai Medical Association Hospital Kagoshima Japan; ^15^ Department of Medical Oncology Okayama Rosai Hospital Okayama Japan; ^16^ Second Department of Surgery University of Occupational and Environmental Health Japan; ^17^ Department of Thoracic Surgery Kanazawa Medical University Ishikawa Japan; ^18^ Department of Respiratory Medicine Wakamatsu Hospital of the University of Occupational and Environmental Health Kitakyushu Japan; ^19^ Department of Respiratory Medicine National Hospital Organization Ureshino Medical Center Saga Japan; ^20^ Department of Medical Oncology Yao Tokushukai General Hospital Osaka Japan; ^21^ Division of Medical Oncology Department of Internal Medicine Teikyo University School of Medicine Tokyo Japan; ^22^ Department of Medical Oncology and Clinical Research National Hospital Organization Yamaguchi‐Ube Medical Center Yamaguchi Japan; ^23^ Department of Thoracic Oncology National Hospital Organization Kyushu Cancer Center Fukuoka Japan; ^24^ Department of Respiratory Medicine Ehime Prefectural Central Hospital Ehime Japan; ^25^ Clinical Research Center Saga University Hospital Saga Japan; ^26^ Education and Research Center for Community Medicine Faculty of Medicine Saga University Saga Japan

**Keywords:** molecular diagnosis, mutations, next‐generation sequencing, non‐small cell lung cancer

## Abstract

**Background:**

*EGFR* mutations are good predictive markers of efficacy of EGFR tyrosine kinase inhibitors (EGFR‐TKI), but whether comprehensive genomic analysis beyond *EGFR* itself with circulating tumor DNA (ctDNA) adds further predictive or prognostic value has not been clarified.

**Methods:**

Patients with NSCLC who progressed after treatment with EGFR‐TKI, and with *EGFR* T790 M detected by an approved companion diagnostic test (cobas^®^), were treated with osimertinib. Plasma samples were collected before and after treatment. Retrospective comprehensive next‐generation sequencing (NGS) of ctDNA was performed with Guardant360^®^. Correlation between relevant mutations in ctDNA prior to treatment and clinical outcomes, as well as mechanisms of acquired resistance, were analyzed.

**Results:**

Among 147 patients tested, 57 patients received osimertinib, with an overall response rate (ORR) of 58%. NGS was successful in 54 of 55 available banked plasma samples; *EGFR* driver mutations were detected in 43 (80%) and T790 M in 32 (59%). The ORR differed significantly depending on the ratio (T790 M allele fraction [AF])/(sum of variant AF) in ctDNA (*p* = 0.044). The total number of alterations detected in plasma by NGS was higher in early resistance patients (*p* = 0.025). T790 M was lost in 32% of patients (6 out of 19) after acquired resistance to osimertinib. One patient with *RB1* deletion and copy number gains of *EGFR*, *PIK3CA*, and *MYC* in addition to T790 M, showed rapid progression due to suspected small cell transformation.

**Conclusions:**

NGS of ctDNA could be a promising method for predicting osimertinib efficacy in patients with advanced NSCLC harboring *EGFR* T790 M.

## Graphical abstract

A retrospective analysis of comprehensive next‐generation sequencing (NGS) of ctDNA in a multi‐center, prospective observational cohort study conducted to examine the efficacy of liquid biopsy as a predictive marker for third generation treatment with the EGFR tyrosine kinase inhibitor (EGFR‐TKI), osimertinib is reported in this paper. We demonstrated that the number of genomic alterations was significantly higher in non‐responders than in responders to osimertinib, suggesting that plasma NGS could be a better method for predicting osimertinib efficacy in patients with advanced NSCLC.

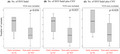

## 1 INTRODUCTION

Liquid biopsy utilizing circulating tumor DNA (ctDNA) has become an accessible, non‐invasive approach for evaluating genomic alterations in advanced stage cancers.^1^, ^2^ Considering tumor evolution in which genomic alternations arise in response to treatment, it is essential to assess emerging genomic alterations that arise after initial therapy so that they may inform decisions about later lines of treatment.^3^ At the time of disease progression, performing tumor genomic assessment using plasma is more convenient than repeating a tumor biopsy. Furthermore, because tumor DNA can be shed by all metastatic tumors within the body, ctDNA analysis may better reflect the global status of a tumor's genomic alterations. Our research group has examined whether genomic alterations of non‐small cell lung cancer (NSCLC) can be detected by ctDNA analysis, and their correlation with tumor progression, starting with the HASAT study that focused on *EGFR* T790 M.^4^ This gatekeeper mutation of *EGFR* occurs in 50%–60% of patients with NSCLC who have *EGFR* activating mutations and who acquire resistance to first and second generation EGFR tyrosine kinase inhibitors (EGFR‐TKI).^5^, ^6^ Thereafter, cobas^®^ EGFR mutation test version 2 was approved in Japan as a companion diagnostic test using tissue or plasma for the detection of T790 M when the physician is considering treatment with the third generation EGFR‐TKI, osimertinib, which is targeted for T790 M as well as *EGFR* activating mutations.^7^ The cobas test is based on allele‐specific real‐time PCR, and the detection limit has been reported to be 0.025%−0.15% by analysis using fragmented DNA isolated from lung cancer cell lines bearing *EGFR* mutations. Because detection of ctDNA is associated with tumor progression, it can also be characterized as a prognostic factor.^4^, ^8^ However, it had not been clarified whether liquid biopsy with ctDNA is useful for assessing treatment efficacy. A phase III trial of osimertinib among patients with NSCLC who had tumors harboring *EGFR* T790 M (AURA 3) clearly demonstrated that liquid biopsy can predict efficacy of osimertinib by revealing T790 M in plasma.^9^ However, level of efficacy varied from complete response to primary resistance even among patients in whom T790 M was detected. We hypothesized that co‐existing genomic alterations beyond *EGFR* might impact treatment efficacy and that comprehensive genomic analysis could lead to more precise prediction of treatment efficacy.

Here we conducted a prospective, multi‐center, observational study to examine the efficacy of liquid biopsy as a predictive marker for the third generation EGFR‐TKI, osimertinib. Using banked plasma samples, we retrospectively performed comprehensive genomic analysis with next‐generation sequencing (NGS) using Gurdant360, a commercially available NGS assay for ctDNA.^10^, ^11^ Our aims were to investigate whether the co‐existence of variants other than T790 M is correlated with response to osimertinib and to assess the clinical utility of NGS with ctDNA for better prediction of treatment efficacy.

## 2 MATERIALS AND METHODS

### 2.1 Study design

This was a retrospective analysis using banked plasma samples collected for the S‐PLAT study, a prospective, multi‐center, observational study to investigate the usefulness of liquid biopsy for predicting the outcome of treatment with third generation EGFR tyrosine kinase inhibitor among patients with advanced NSCLC whose disease progressed after treatment with first or second generation EGFR‐TKI. Eligible patients were those with NSCLC having *EGFR* activating mutations—including G719X, exon 19 deletion, L858R, and L861Q—whose diseases had progressed after treatment with first or second generation EGFR‐TKI. Patients were excluded if they were treated with cytotoxic chemotherapy within 14 days of the first dose of study treatment or if they had radiotherapy within 4 weeks of the first dose of study treatment. Patients having a history of treatment with osimertinib or immune checkpoint inhibitors were also excluded. The patients in whom T790 M was confirmed by an approved companion diagnostic test, cobas^®^ *EGFR* Mutation Test v2, were treated with osimertinib. The specimens tested by cobas were tissue, plasma, or both, depending on each physician's choice. Comprehensive molecular analysis was performed with Guardant360 on ctDNA extracted from plasma collected before osimertinib treatment and again at the time of disease progression. The primary objective of this study was to determine whether tumor responses, such as overall response rate (ORR) and progression‐free survival (PFS) under osimertinib among patients who are positive for T790 M in ctDNA, using the mutation‐biased polymerase chain reaction (PCR) and quenched probe (MBP‐QP) method (a highly sensitive mutation system developed in our laboratory), are equivalent to those from historical data based on cobas testing of tumor tissue in the AURA study.^12^, ^13^ In this paper, we focused on the exploratory objectives, which were to assess the association of ORR and PFS to allele fraction (AF) of T790 M or other variants detected by NGS with ctDNA. Response evaluation by imaging was recommended every 8 weeks, and performed according to the Response Evaluation Criteria in Solid Tumors (RECIST) ver.1.1. This study was conducted in accordance with the Declaration of Helsinki and approved by the ethics committees of all participating facilities represented by Saga University. Written informed consent was obtained from all participants. The study was registered at UMIN‐CTR (UMIN000025930).

### 2.2 Molecular analysis with ctDNA

From each patient, prior to the start of osimertinib treatment, 10 ml of peripheral blood was collected into a blood tube containing 3.8% citrate. Blood was centrifuged at 3,000 rpm for 20 min at 4℃ to collect 4 ml of plasma, and ctDNA was extracted with a Maxwell RSC® ccfDNA plasma kit (Promega, WI, USA).^14^, ^15^ At the time of disease progression, peripheral blood was collected in two 10 ml Cell‐Free DNA BCT® tubes (Streck, NE, USA). Extracted ctDNA or peripheral blood was shipped for Guardant360 analysis (Guardant Health Inc., CA, USA). The cobas plasma test was performed by designated testing companies (SRL Inc., Tokyo, Japan; LSI Medience Corporation, Tokyo, Japan; BML Inc., Tokyo, Japan).

### 2.3 Statistical analysis

The association between treatment efficacy with osimertinib and *EGFR* mutation AF by NGS was tested with Pearson's χ^2^ test. The survival rate was calculated according to the Kaplan–Meier method and the log‐rank test was used for assessing differences. The comparison between early resistance and non‐early resistance on clinical and genomic parameters was tested with the χ^2^ test for categorical data and the nonparametric Mann–Whitney U test for continuous data. For multivariable analysis, a logistic regression model was applied with explanatory variables that were statistically significant (*p* ≤ 0.20) in the two‐group comparison test. Odds ratios (OR) with 95% confidence intervals (CI) were estimated. The AF difference between pre‐treatment and after progressive disease was assessed with the nonparametric Wilcoxon signed‐rank test. Statistical significance was declared if *p* < 0.05. Statistical analysis was conducted with SPSS version 19 (IBM SPSS Statistics, IBM, Tokyo, Japan).

## 3 RESULTS

### 3.1 Study flow and patient characteristics

The flow of this study is shown in Figure S1. Eligible patients were registered from 28 Japanese hospitals between February 2017 and January 2019. Although 153 participants were enrolled, 6 of them were withdrawn due to worsening general condition or difficulty with tissue sampling. Samples from the remaining 147 patients underwent cobas analysis with tissue re‐biopsy (n = 72), ctDNA (n = 60), or both (n = 15) as companion diagnostics for osimertinib (Figure S2A), and 60 patients were shown to harbor T790 M. T790 M was detected in 52.9% and 24.0% with tissue and ctDNA samples, respectively, using cobas (*p* = 0.0002, χ2 test; Figure S2B). Three patients were not treated with osimertinib because they declined treatment or met one of the exclusion criteria, such as hepatitis B antigen positivity, leaving 57 patients who were treated with osimertinib (Table 1). During the follow‐up period, 36 patients’ diseases progressed during osimertinib treatment. The median age of all 57 osimertinib‐treated patients was 72 (range 42–88) years, and the majority were female (68%), had never smoked (74%), and had stage IV or recurrent tumors after surgery (84%). Extrathoracic metastases were observed among 53% of the osimertinib‐treated patients. *EGFR* activating mutations included exon 19 deletion among 53% and L858R among 47%. T790 M detected by cobas with plasma was found among 16 (28%) and with tumor tissue among 44 (77%), including 3 patients with T790 M detected in both tissue and plasma. Fifty‐six patients were evaluated for response to osimertinib; one patient developed a cerebral infarction (unrelated to treatment) and could not be evaluated. Among all patients treated, the ORR to osimertinib was 58% (33 of 57), with disease control rate (DCR) 91% (52 of 57). Median PFS was 14 months (95% CI 9.863–18.137) and median follow‐up period was 24 months (range 11–35).

**TABLE 1:** Characteristics of osimertinib‐treated patients in this study

Total	N = 57
Age, median (range)	72 (42–88)
Sex	
Female	39 (68%)
Male	18 (32%)
Smoking status	
Never smoker	42 (74%)
Ex or current smoker	15 (26%)
Stage	
Ⅲ	9 (16%)
Ⅳ or recurrence	48 (84%)
Extrathoracic metastasis	
Present	30 (53%)
Absent	27 (47%)
*EGFR* activating mutation	
Ex19 del	30 (53%)
L858R	27 (47%)
T790 M detection (cobas^®^)^a^	
Tissue	44 (77%)
ctDNA	16 (28%)
Best response to osimertinib^b^	
CR	3 (5%)
PR	30 (53%)
SD	19 (33%)
PD	4 (7%)
NE	1 (2%)

^a^ The sample for T790 M test was each clinician's choice. T790 M was detected in both tissue and ctDNA in three patients.

^b^ One patient was not evaluated for response to osimertinib.

### 3.2 NGS analysis with ctDNA before treatment with osimertinib

**FIGURE 1:**
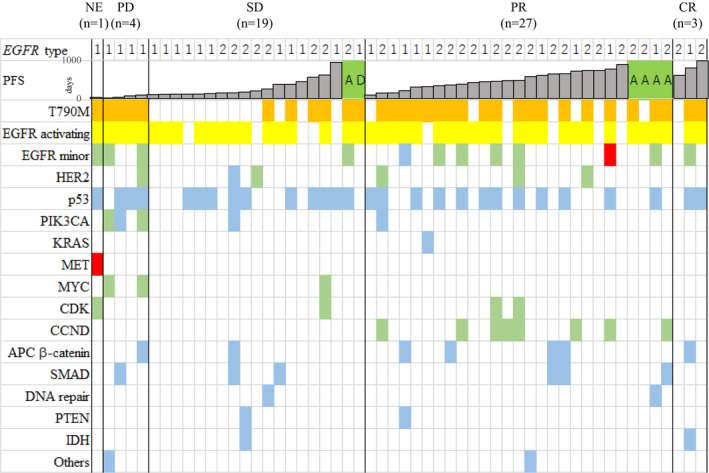
Molecular profile of circulating tumor DNA in plasma samples from patients prior to osimertinib treatment. Fifty‐four patients are shown among whom NGS was successful, from among 57 patients treated with osimertinib, including 3 CR, 27 PR, 19 SD, and 4 PD patients. One patient could not be evaluated for tumor response because of accidental development of cerebral infarction. Single‐nucleotide variants or insertions/deletions deemed to be oncogenic, likely oncogenic, or predicted oncogenic by OncoKB are included. NE, not evaluated; PD, progressive, disease; SD, stable disease; PR, partial response; CR, complete response; SNV/Indel, single‐nucleotide variants and/or insertion/deletions; CNV, copy number variants. In *EGFR* type, 1 and 2 indicate L858R and exon19 deletions, respectively. 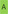, discontinuation by adverse effects; 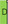, discontinuation at the request of the patient; 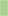, CNV; 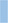, SNV/Indel; 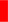, both CNV and SNV/Indel existed

**FIGURE 2:**
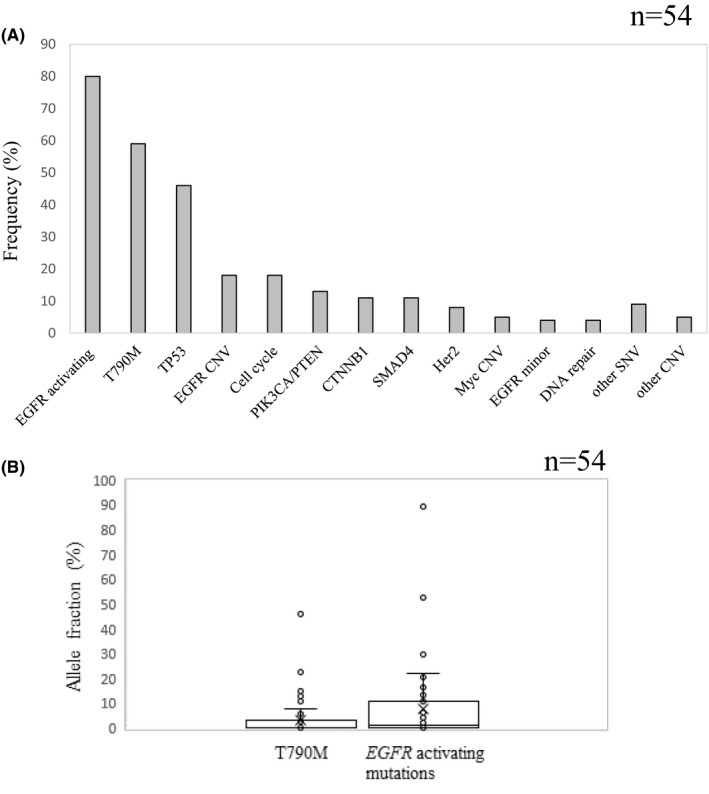
(A) Frequency of variants detected with ctDNA before treatment with osimertinib. (B) Allele fraction of T790 M and *EGFR* activating mutations with ctDNA before treatment with osimertinib. SNV, single‐nucleotide variant; CNV, copy number variant

To investigate the potential influence of oncogenic mutations in addition to *EGFR*, we analyzed, with a comprehensive NGS platform (Guardant360), 55 available plasma samples from 57 patients, where the samples had been banked before treatment with osimertinib (Figure S1). NGS was technically successful in 54 out of the 55 samples (98%), and ctDNA was detected in 52 of the 54 samples that had complete analysis (96% detection rate). The findings of each patient are summarized in Figure 1. Synonymous mutations and variants of unknown significance (VUS) were excluded. In addition to *EGFR* single‐nucleotide variants (SNV), copy number variants (CNV) in *EGFR*, *ERBB2*, and cell cycle‐related genes were detected along with SNV and insertions/deletions (Indels) in genes such as *TP53*, *PIK3CA*, *CTNNB1*, and *SMAD4*. *EGFR* driver mutations were found in 43 (80%) and T790 M in 32 (59%) of the 54 samples (Figure 2A). The median AF of T790 M and *EGFR* activating mutations was 0.216 (0–45.78) and 1.05 (0–89.6), respectively (Figure 2B).

**FIGURE 3:**
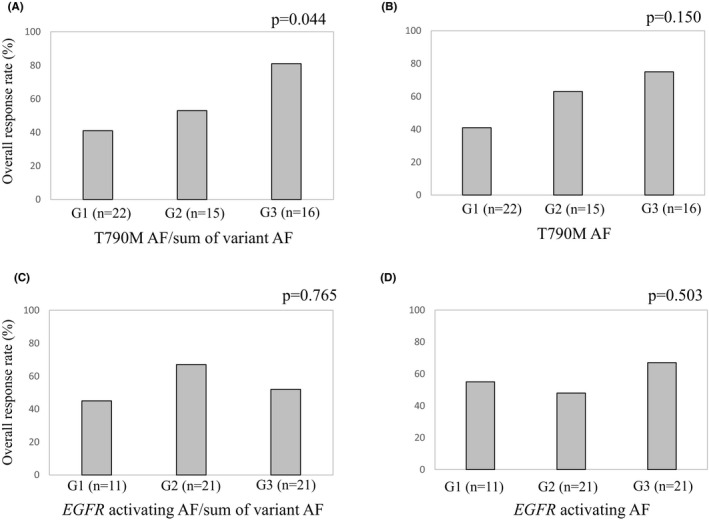
The overall response rate is shown depending on (A) (T790 M allele fraction (AF))/(sum of variant AF), (B) T790 M AF, (C) (*EGFR* activating mutations AF)/(sum of variant AF), and (D) *EGFR* activating mutations AF in ctDNA. Patients were divided into three groups: G1 (not detected), G2 (below median), and G3 (median and above). One patient was not evaluated for tumor response, so 53 patients were analyzed. The association with overall response rate of osimertinib was tested with Pearson's χ2 test

**FIGURE 4:**
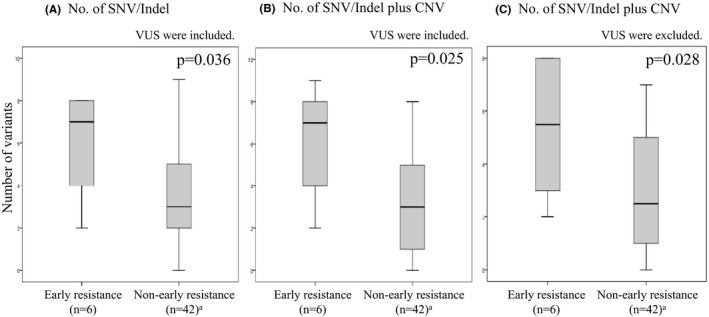
The relationship between the number of genomic alterations detected prior to treatment with osimertinib and the therapeutic response to osimertinib is shown. Six patients, out of 54 among whom NGS was technically successful, were excluded from the analysis because of discontinuation due to adverse effects or patient's request. (A) The number of oncogenic SNVs and Indels including VUS. (B) The sum of SNVs/indels and CNV including VUS. (C) The sum of SNVs/indels and CNV excluding VUS. Non‐responders and responders had PFS of 90 days or less, and more than 90 days, respectively. Comparisons between non‐responders and responders were made with the nonparametric Mann–Whitney U test. SNV/Indel, single‐nucleotide variants and/or insertion/deletions; CNV, copy number variants; VUS, variants of unknown significance

Patients were divided into three groups according to pre‐treatment level of T790 M AF: G1 (not detected), G2 (below median), and G3 (median and above). The ORR were 41% (9 of 22), 63% (10 of 15), and 75% (12 of 16) in G1, G2, and G3, respectively, but the trend was not statistically significant (*p* = 0.150, Figure 3B). However, when grouping was based on the ratio (T790 M AF)/(sum of variant AF), ORR increased significantly according to group: G1 (41%, 9/22), G2 (53%, 8/15), and G3 (81%, 13/16) (*p* = 0.044, Figure 3A). Analysis of *EGFR* activating mutation AF or the ratio (*EGFR* activating mutation AF)/(sum of variant AF) showed no statistically significant correlation with ORR (Figure 3C and D). In a comparison of PFS between patients who were T790 M positive and negative with ctDNA at baseline, a statistically significant difference was not observed (median PFS of T790 M positive: 14 M [95% CI 4.674–23.326]; median PFS of T790 M negative: 22 M [95% CI 0–17.937]; *p* = 0.357; Figure S3). In a comparison of PFS by grouping based on the ratio (T790 M AF)/(sum of variant AF), a statistically significant difference was also not observed (*p* = 0.582, Figure S4). We further analyzed the outcomes that were based on clinical responses to osimertinib, with patients classified as “non‐early resistance” (PFS >90 days) or “early resistance” (PFS ≤90 days), because the range of PFS among the PD patients whose PFS was 15–90 days. Figure 4 shows the relationship between the number of genomic alterations detected prior to treatment with osimertinib and the therapeutic response to osimertinib. The number of oncogenic SNVs and Indels (including VUS) and that plus CNV were both higher in “early resistance” patients, with statistically significant differences (*p* = 0.036 and *p* = 0.025, respectively, Figure 4A, B). Even with VUS excluded, the sum of SNVs/indels and CNVs was higher in “early resistance” than in “non‐early resistance” patients (*p* = 0.028, Figure 4C). When “early resistance” and “non‐early resistance” patients were compared in terms of various clinical factors, the number of oncogenic SNVs and Indels (including VUS), with or without inclusion of CNVs, and *EGFR* activating mutation type showed statistically significant differences between them (Table 2). Multivariable analysis of the association of “early resistance” to osimertinib showed a statistically significant association with SNV/Indels plus CNVs (Table 3).

**TABLE 2:** Comparison between early resistance and non‐early resistance to osimertinib

	Early resistance (n = 6)^a^	Non‐early resistance (n = 42)^a^	*p*
70.5 (58–81)	71.0 (57–85)	0.943	
Sex			
Female	6 (17%)	29 (83%)	0.108
Male	0	13 (100%)	
Smoking status			
Never smoker	6 (17%)	30 (83%)	0.318
Ex or current smoker	0	12 (100%)	
Stage			
Ⅲ	0	7 (100%)	0.578
Ⅳ or recurrence	6 (15%)	35 (85%)	
Extrathoracic metastasis			
Present	5 (19%)	21 (81%)	0.195
Absent	1 (5%)	21 (95%)	
*EGFR* activating mutation			
Ex19 del	0	23 (100%)	0.010
L858R	6 (24%)	19 (76%)	
T790 M AF, median (range)	0.055 (0–45.78)	0.019 (0–22.5)	0.384
T790 M AF/Total AF, median (range)	0.355 (0–0.227)	0.08 (0–0.414)	0.704
*TP53* SNV			
Present	5 (19%)	21 (81%)	0.199
Absent	1 (5%)	21 (95%)	
SNV/Indel plus CNV, median (range)	7 (2–9)	3 (0–8)	0.025

Abbreviations: AF, allele fraction; CNV, copy number variants; SNV/Indel, single‐nucleotide variants and/or insertion/deletions.

^a^ Six patients, out of 54 among whom NGS was technically successful, were excluded from the analysis because of discontinuation due to adverse effects or patient's request. Patient characteristics in early resistance and non‐early resistance were compared with the χ^2^ test for categorical data and the nonparametric Mann–Whitney U test for continuous data.

**TABLE 3:** Multivariable analysis of association with early resistance to osimertinib

	Early resistance (n = 6)^a^	Non‐early resistance (n = 42)^a^	OR (95% CI)	*p*
Extrathoracic metastasis				
Absent	1	21	1.00 (Reference)	
Present	5	21	7.01 (0.216–227.834)	0.273
TP53 SNV				
Absent	1	21	1.00 (Reference)	
Present	5	21	1.868 (0.068–51.171)	0.711
SNV/Indel plus CNV	7 (2–9)	3 (0–8)	2.170 (1.078–4.368)	0.03

Abbreviations: CNV, copy number variants; SNV/Indel, single‐nucleotide variants and/or insertion/deletions.

^a^ Six patients, out of 54 among whom NGS was technically successful, were excluded from the analysis because of discontinuation due to adverse effects or patient's request.

### 3.3 Analysis of acquired resistance to osimertinib based on NGS with ctDNA

**FIGURE 5:**
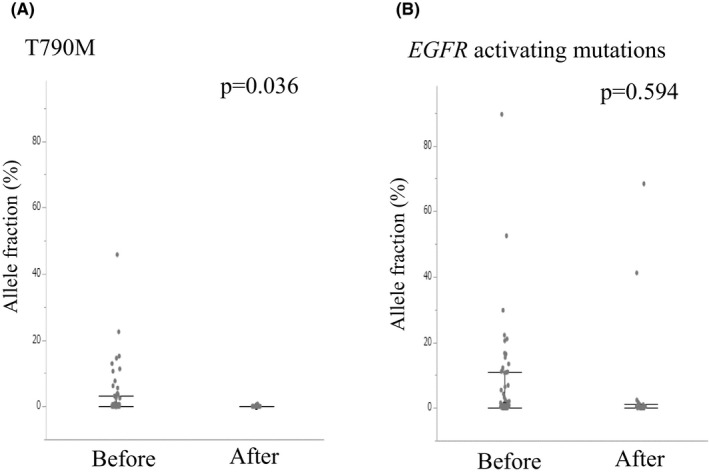
Difference between before treatment with osimertinib and after disease progression for AF of T790 M (A) and *EGFR* activating mutations (B). Nineteen patients were analyzed among whom NGS was successful after PD. AF, allele fraction. *P* values were calculated by Wilcoxon signed‐rank test

NGS analysis was also performed after disease progression under osimertinib treatment and compared to the NGS profiles before treatment in order to assess acquired resistance mechanisms. Plasma samples could be collected and NGS successfully performed in 20 of the 36 patients who developed disease progression during osimertinib treatment; variants were detected in 19 of these 20 patients. The AF of T790 M was significantly lower after disease progression, but not that of *EGFR* activating mutations (*p* = 0.036 and *p* = 0.594, respectively; Figure 5). In 18 of 19 cases, T790 M was not detected after disease progression, and T790 M was neither detected before treatment nor after disease progression in 10 cases. Table 4 shows the frequencies of T790 M loss (change from presence to absence) and variants newly observed. T790 M was lost in 37% of patients (7 out of 19). New alterations included *EGFR* minor mutations (S752C and S306L) in two patients, *MET* SNV/CNV in two patients, *TP53* SNV in three patients, *PIK3CA* SNV in two patients, cell cycle‐related genes SNV/CNV in two patients, and *MYC* CNV in one patient. Other variants included SNVs in the genes *NF1, CDK12, ARID1A, CTNNB1, DDR2,* *and APC*, and CNVs of *CDK6, CCNE1,* *FGFR1*, and *BRAF*. There was no case with *EGFR* C797S.

**TABLE 4:** Variants detected after PD to osimertinib n = 19

Variants	Number of patients
T790 M loss	7 (37%)
EGFR minor mutations	2 (11%)
MET SNV and/or CNV	2 (11%)
TP53 SNV	3 (16%)
PIK3CA SNV	2 (11%)
MYC CNV	1 (5%)
Cell cycle‐related SNV and/or CNV	2 (11%)
Others	4 (21%)

Abbreviations: CNV, copy number variant; SNV, single‐nucleotide variant.

**FIGURE 6:**
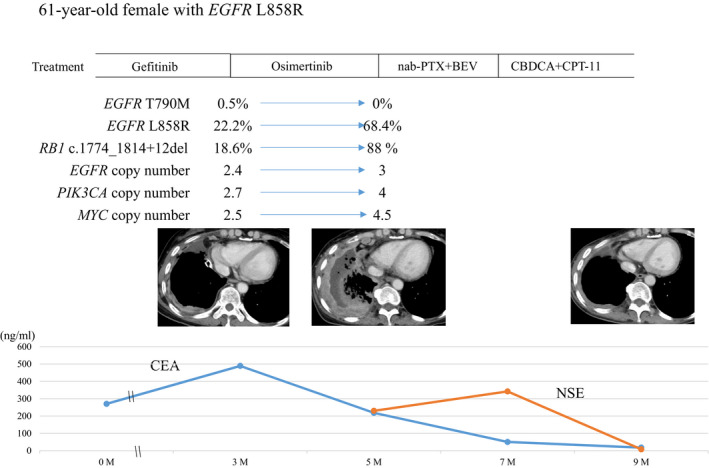
A case of possible small cell transformation. History of treatment and change of variants detected by NGS analysis using Guardant360 are shown with change of CT images and tumor markers such as CEA and NSE. nab‐PTX, nanoparticle albumin‐bound paclitaxel. BEV, bevacizumab; CBDCA, carboplatin; CPT‐11, irinotecan; CEA, carcinoembryonic antigen; CT, computed tomography; NSE, neuron‐specific enolase

We also observed a patient with possible small cell transformation, with suspicion triggered by the finding from ctDNA NGS profiling (Figure 6). This patient, a 61‐year female non‐smoker with *EGFR* L858R, was treated with osimertinib after disease progression on gefitinib, her fifth line of treatment for NSCLC. Prior to osimertinib treatment, the NGS assay detected *EGFR* L858R (AF 22.2%) and T790 M (AF 0.5%) along with *RB1* c.1774_1814+12del (AF 18.6%) and copy number gains of *EGFR*, *PIK3CA*, and *MYC*, but no *TP53* mutation. Shortly after the patient was started on osimertinib, the tumor rapidly progressed. Although *EGFR* T790 M became undetectable, AFs of both *EGFR* L858R and the *RB1* indel increased. Her physician suspected small cell transformation, but a tissue biopsy could not be obtained because of the patient's rapid deterioration. Neuron‐specific enolase (NSE) was found to be elevated in serum, and a regimen for small cell carcinoma (carboplatin plus irinotecan) was initiated. This regimen caused shrinkage of the right pleural dissemination along with a decrease in NSE. Brain metastasis occurred after three courses of the regimen and was followed by deterioration of the pleural dissemination. Despite treatment with the anti‐PD‐L1 antibody atezolizumab, the patient died 2 months later.

## 4 DISCUSSION

Osimertinib for treatment of NSCLC is a potent EGFR‐TKI for previously treated patients as well as in the first‐line setting.^16^, ^17^, ^18^ However, some tumors demonstrate primary resistance: 6% among previously treated patients and 1% among those treated in the first line. *EGFR* activating mutations are known to be strong tumor drivers, but in addition to *EGFR* mutations, a diversity of genomic profiles has been reported recently on the basis of multi‐region whole‐exome sequencing.^19^ Genomic variants outside of EGFR may impact treatment efficacy of EGFR‐TKI. Recent reports based on NGS showed that *STK11* mutation co‐existing with *KRAS*‐mutated NSCLC caused refractory response of immune checkpoint inhibitors (ICI).^20^ In patients with prostate cancer, *BRCA2* and *ATM* defects, TP53 mutations, and AR gene structural rearrangements were strongly associated with poor clinical outcome of patients treated with androgen receptor‐directed therapies.^21^ Considering these results, we hypothesized that co‐existence of *EGFR* mutations with other variants might cause primary resistance to, or weak efficacy of, EGFR‐TKI.

At first, we evaluated the relationship between T790 M AF and treatment efficacy of osimertinib. Since osimertinib shows more potent inhibition against T790 M than against *EGFR* activating mutations,^22^ we expected that osimertinib would have greater efficacy in cases with high T790 M AF, but the results showed no significant difference. However, T790 M AF divided by the sum of AF of total variants was significantly associated with ORR. The amount of ctDNA has been known to be associated with tumor burden or distant metastasis.^4^, ^8^ Therefore, it is assumed that T790 M AF corrected by total AF could represent the proportion of T790 M among whole tumor in each patient. A similar result has been reported in that T790 M purity shown by the ratio of T790 M AF to maximum somatic AF was associated with osimertinib efficacy.^23^ However, our results showed that the relationship between the (T790 M AF)/(maximum variant AF) proportion and ORR was not statistically significant (*p* = 0.10, data not shown). In this study, NGS using Guardant360 revealed various co‐existing genomic alterations prior to osimertinib treatment, possibly because many patients had received several treatment regimens prior to enrolling. The number of genomic alterations (the sum of SNVs and indels with or without CNVs) was significantly higher in “early resistance” patients. One explanation for these results is that additional variants arising after modification of previous treatments might cause activation of alternative pathways or cross talk with the main pathway. Even in the early stages of lung cancer among patients with *EGFR* mutations, a high number of truncal mutations and overall mutation burden were significantly related to shorter overall survival.^19^ However, we could not find any reports about the relationship between efficacy of treatment including osimertinib and the number of whole variants in addition to *EGFR* mutations. TP53 mutation has been reported to impact clinical outcome by facilitating genomic instability,^19^ but we did not observe any relationship between response to osimertinib and specific co‐occurring variants in genes such as *TP53* or *PIK3CA*. According to Jaml‐Hanjani's report on tumor evolution of NSCLC, genomic doubling caused intra‐tumor heterogeneity of copy number alterations and mutations, and that was associated with poor outcome.^3^ The NSCLC patients with high copy number alterations observed in subclonal trajectory showed shorter disease‐free survival than those with low copy number. Therefore, the number of variants, rather than the presence of specific alterations, could be associated with impaired treatment efficacy. As shown in the present paper, recent technology has enabled evaluation of total variants including SNVs and CNVs with ctDNA, and it is worth investigating the association between total variants measured with ctDNA and efficacy of EGFR‐TKIs such as osimertinib.

Mechanisms of acquired resistance to osimertinib are known to be heterogenous, including *EGFR* C797X; loss of T790 M; SNV of *PIK3CA*, *KRAS*, and *BRAF*; and amplification of *MET*.^24^, ^25^ In addition to these variants, epithelial‐to‐mesenchymal transition, manifested as small cell carcinoma (SCLC) transformation, is not an infrequent cause of EGFR‐TKI resistance. In our cohort, we detected one patient whose cancer we suspected of being SCLC transformation, on the basis of tumor markers and treatment efficacy of a regimen for SCLC; however, it could not be confirmed by pathological analysis, which is necessary for diagnosis.^26^, ^27^, ^28^, ^29^ Molecular analysis with whole‐genome sequencing has shown that inactivation of *RB1* and *TP53* can be observed in advanced stage *EGFR*‐mutated NSCLC. *RB1* loss is known to occur in 100% of SCLC transformation cases, and *MYC* amplification is associated with poor prognosis.^29^, ^30^, ^31^ In our suspected case of SCLC transformation, we detected both *RB1* deletion and *MYC* amplification before treatment with osimertinib, and these alterations expanded because of resistance to osimertinib. A further benefit of molecular analysis with ctDNA is, therefore, that it might raise the clinical suspicion of SCLC transformation, triggering a tissue biopsy to guide appropriate therapy.

In patients with NSCLC whose disease has progressed during first or second generation EGFR‐TKI treatment, detection of *EGFR* T790 M supports the decision to initiate osimertinib treatment. As the quality of ctDNA detection methodologies has improved, most patients with disease progression no longer require re‐biopsy for evaluation of actionable tumor biomarkers. One limitation of our study is the small study group size; another is that tumor response and PFS were assessed by the investigators, not by independent central reviewers. In addition, our study was limited by the fact that ctDNA analysis of *EGFR* alterations was conducted in a population of patients with known T790 M, but no cases with wild‐type EGFR, and therefore does not reflect the clinical setting where there is no prior information regarding mutations of interest. However, our study demonstrates that the plasma‐based comprehensive genomic panel is a practical tool for precise prediction of treatment efficacy by detection of total number of variants in addition to driver mutations, and for analysis of potential mechanisms of resistance to TKIs. Although osimertinib treatment is still recommended for patients with *EGFR* activating mutations and T790 M, we can be prepared to change treatment strategy quickly for patients suspected of being early resistance. In addition, the pathological significance of co‐existent variants with *EGFR* mutations needs to be investigated, leading to a new treatment strategy in combination with EGFR‐TKI. A further clinical trial using plasma NGS could confirm an expanded role for ctDNA to allow better identification of patients with NSCLC who are most likely to benefit (or patients who are most likely not to benefit) from targeted therapies such as osimertinib.

## CONFLICT OF INTEREST

Dr. Sueoka‐Aragane reports grants from AstraZeneca, during the conduct of the study; grants and personal fees from Taiho Pharmaceutical, grants and personal fees from Chugai Pharmaceutical, grants and personal fees from Boehringer Ingelheim, grants and personal fees from Eli Lilly, grants from Ono Pharmaceutical, personal fees from AstraZeneca, outside the submitted work. Dr. Nakashima has nothing to disclose. Dr. Yoshida has nothing to disclose. Dr. Matsumoto has nothing to disclose. Dr. Iwanaga has nothing to disclose. Dr. Ebi has nothing to disclose. Dr. Nishiyama has nothing to disclose. Dr. Yatera reports grants from Pfizer, grants from Shionogi, grants from Sumitomo Dainippon Pharma, grants and personal fees from Chugai Pharmaceutical, grants from MSD, grants from Teijn, grants from GlaxoSmithKline, personal fees from Ono Pharmaceutical, personal fees from Taiho Pharmaceutical, personal fees from Eli Lilly, personal fees from AstraZeneca, personal fees from Novartis, personal fees from Toa Eiyo, personal fees from Asahi Kasei Pharma, personal fees from Kyowa Kirin, personal fees from Daiichi Sankyo, personal fees from Nippon Boehringer Ingelheim, personal fees from Astellas Pharma, outside the submitted work. Dr. Kuyama has nothing to disclose. Dr. Fukuda reports grants from AstraZeneca, grants from Eli Lilly, grants from MSD, personal fees from Ono Pharmaceutical, personal fees from MSD, personal fees from Chugai Pharmaceutical, outside the submitted work. Dr. Ushijima has nothing to disclose. Dr. Umeguchi has nothing to disclose. Dr. Harada reports grants from Eli Lilly, grants and personal fees from MSD, grants and personal fees from Chugai Pharmaceutical, grants from Pfizer, grants and personal fees from Bristol‐Meyers Squib, grants from AstraZeneca, grants from Novartis, grants from Kissei, grants from Takeda, personal fees from Ono Pharmaceutical, personal fees from Kyorin, personal fees from Daiichi Sankyo, outside the submitted work. Dr. Kashiwabara reports personal fees from AstraZeneca, personal fees from Chugai Pharmaceutical, personal fees from TAIHO Pharmaceutical, personal fees from Nippon Boehringer Ingelheim, personal fees from Ono Pharmaceutical, personal fees from Eli Lilly, personal fees from Merck Sharp & Dohme, outside the submitted work. Dr. Suetsugu has nothing to disclose. Dr. Fujimoto reports grants from KISSEI, grants from MSD, personal fees from ONO, personal fees from Bristol‐Meyers Squib, personal fees from Kyorin, outside the submitted work. Dr. Tanaka reports grants and personal fees from Taiho Pharmaceutical, grants and personal fees from Chugai Pharmaceutical, grants and personal fees from Boehringer Ingelheim, grants and personal fees from Eli Lilly, grants and personal fees from Ono Pharmaceutical, personal fees from MSD, personal fees from Bristol‐Meyers Squib, personal fees from Covidien Japan, personal fees from Johnson & Johnson, personal fees from AstraZeneca, outside the submitted work. Dr. Uramoto reports grants from Daiichi Sankyo, grants from Shionogi, grants and personal fees from Boehringer Ingelheim, grants and personal fees from Chugai Pharmaceutical, grants and personal fees from Taiho Pharmaceutical, grants from MSD, grants and personal fees from Eli Lilly, grants from Astellas Pharma, grants from Pfizer, personal fees from AstraZeneca, outside the submitted work. Dr. Yoshii reports grants from Shionogi Pharmaceutical, outside the submitted work. Dr. Nakatomi has nothing to disclose. Dr. Koh has nothing to disclose. Dr. Seki reports grants and personal fees from Eli Lilly, grants and personal fees from Chugai Pharmaceutical, grants and personal fees from Taiho Pharmaceutical, grants and personal fees from Pfizer Japan, grants and personal fees from Ono Pharmaceutical, grants and personal fees from Nippon Boehringer Ingelheim, personal fees from AstraZeneca, personal fees from MSD Oncology, personal fees from Bristol Myers Squibb Japan, outside the submitted work. Dr. Aoe has nothing to disclose. Dr. Nosaki reports personal fees from MSD, personal fees from Nippon Kayaku, personal fees from AstraZeneca, personal fees from Bristol Myers Squibb, personal fees from Chugai Pharmaceutical, personal fees from Eli Lilly, personal fees from Pfizer, personal fees from Taiho Pharmaceutical, outside the submitted work. Dr. Inoue has nothing to disclose. Dr. Takamori has nothing to disclose. Dr. Kawaguchi has nothing to disclose.

## Supporting information

Fig S1‐S4

## Data Availability

The data that support the findings of this study are available from the corresponding author upon reasonable request. Anonymized data will be made available on request in ac‐ cordance with institutional policies.
