# Supplementary material for: The role of comprehensive analysis with circulating tumor DNA in advanced non‐small cell lung cancer patients considered for osimertinib treatment
Source: Cancer Med. 2021 May 12;10(12):3873–85. doi: 10.1002/cam4.3929 (PMC8209625; doi:10.1002/cam4.3929)
Supplement: Supplementary file 1 — Fig S1‐S4 [file CAM4-10-3873-s001.pptx]

## Slide 1
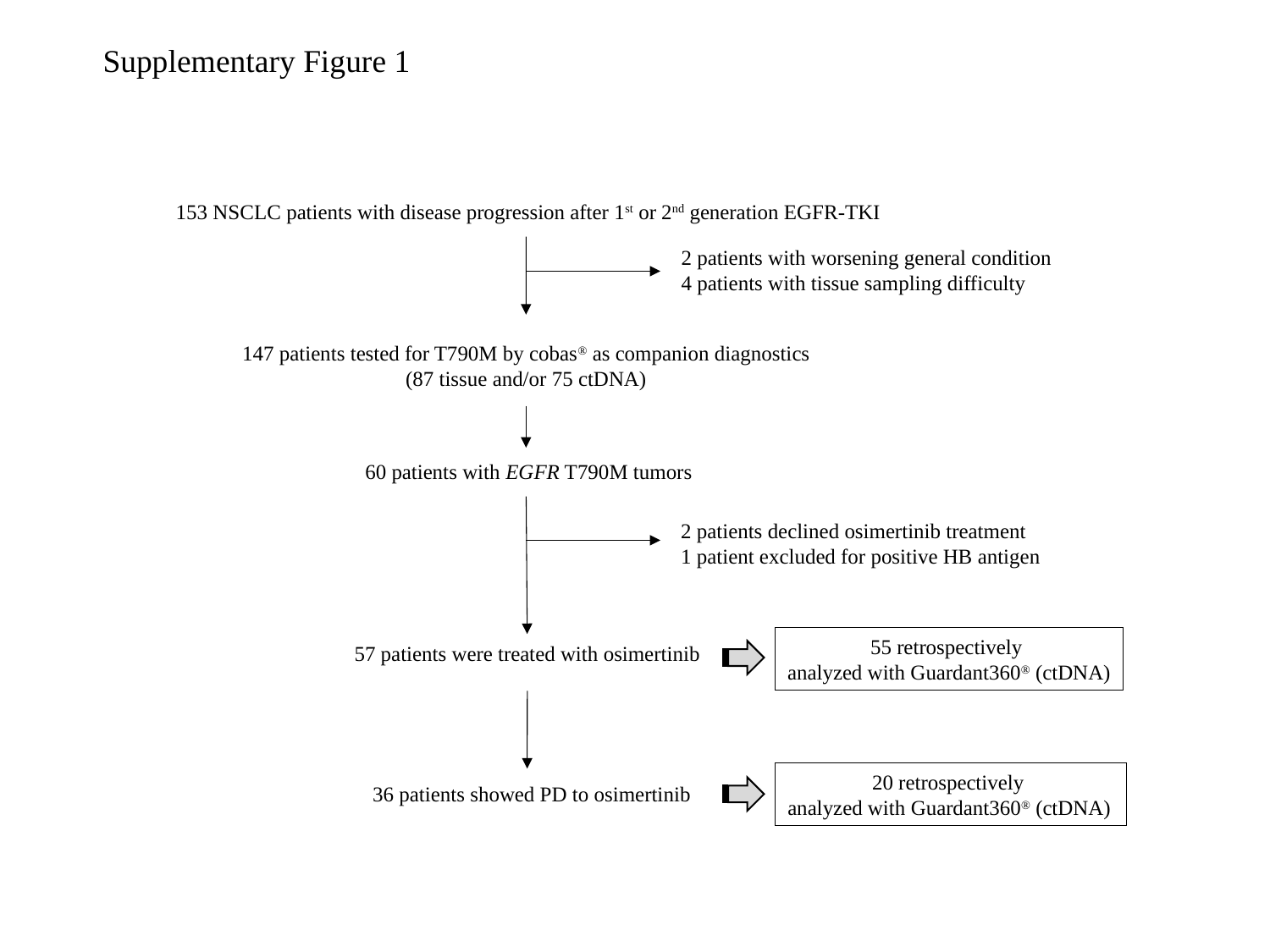

Supplementary Figure 1
153 NSCLC patients with disease progression after 1st or 2nd generation EGFR-TKI
2 patients with worsening general condition
4 patients with tissue sampling difficulty
147 patients tested for T790M by cobas® as companion diagnostics
(87 tissue and/or 75 ctDNA)
60 patients with EGFR T790M tumors
 2 patients declined osimertinib treatment
 1 patient excluded for positive HB antigen
55 retrospectively
analyzed with Guardant360® (ctDNA)
57 patients were treated with osimertinib
20 retrospectively
analyzed with Guardant360® (ctDNA)
36 patients showed PD to osimertinib

## Slide 2
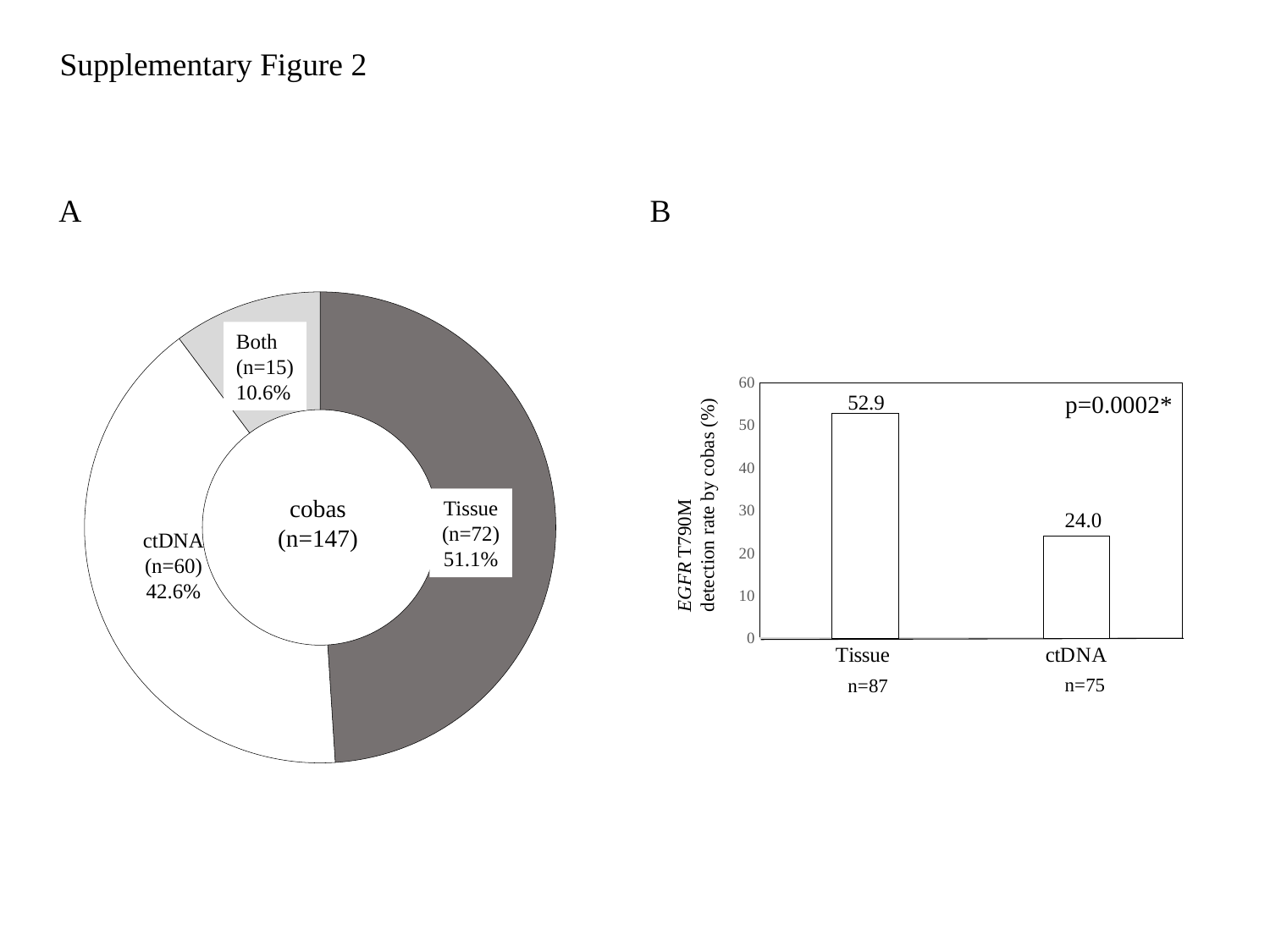

Supplementary Figure 2
A
B
### Chart
| Category | |
|---|---|
| 組織 | 72.0 |
| 血漿 | 60.0 |
| 両方 | 15.0 |Both
(n=15)
10.6%
cobas
(n=147)
Tissue
(n=72)
51.1%
ctDNA
(n=60)
42.6%
### Chart
| Category | T790M陽性率 |
|---|---|
| Tissue | 52.9 |
| ctDNA | 24.0 |52.9
p=0.0002*
EGFR T790M
detection rate by cobas (%)
24.0
n=75
n=87

## Slide 3
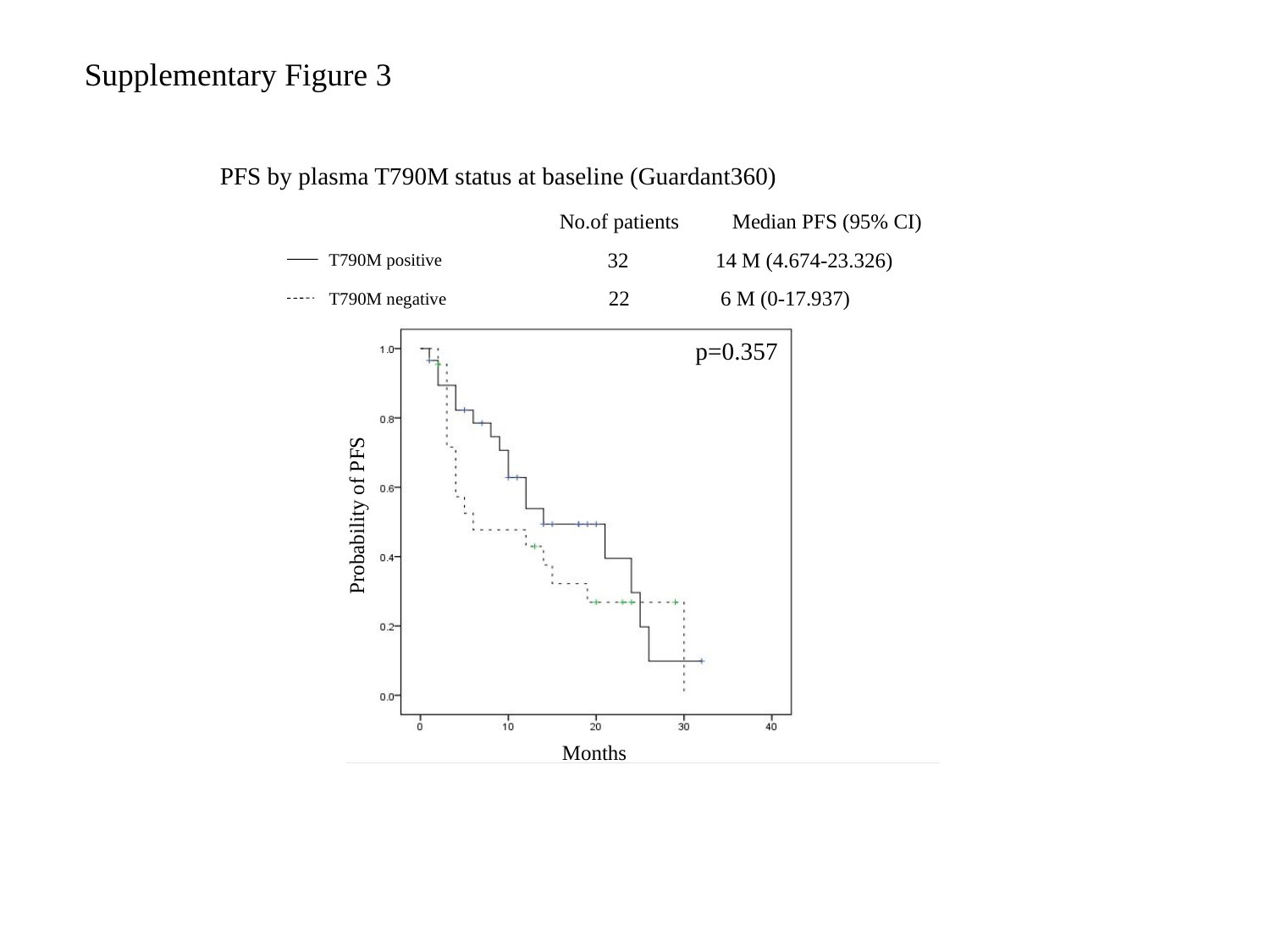

Supplementary Figure 3
PFS by plasma T790M status at baseline (Guardant360)
No.of patients
Median PFS (95% CI)
32
14 M (4.674-23.326)
T790M positive
22
6 M (0-17.937)
T790M negative
p=0.357
Probability of PFS
Months

## Slide 4
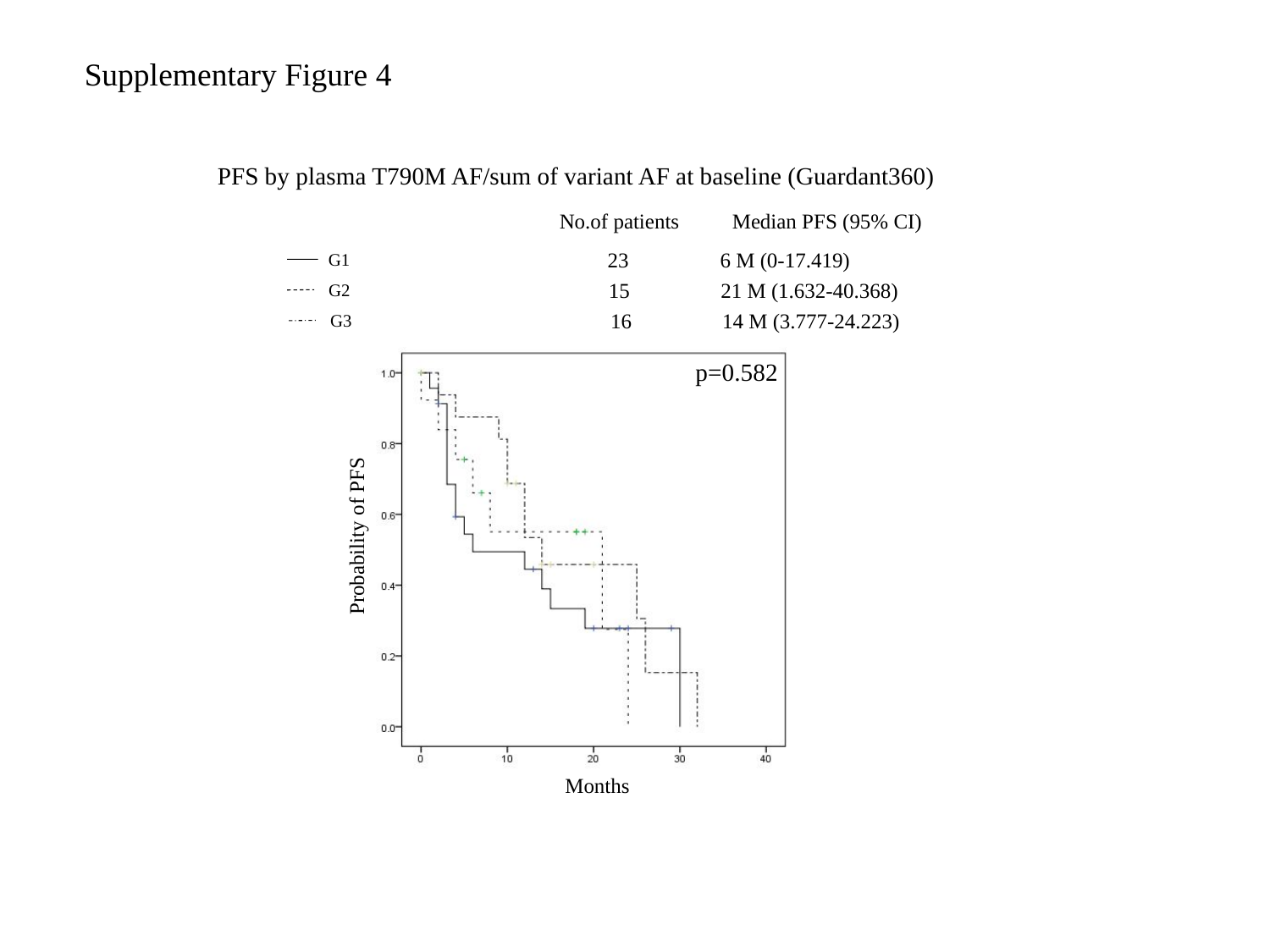

Supplementary Figure 4
PFS by plasma T790M AF/sum of variant AF at baseline (Guardant360)
No.of patients
Median PFS (95% CI)
23
6 M (0-17.419)
G1
15
21 M (1.632-40.368)
G2
16
14 M (3.777-24.223)
G3
p=0.582
Probability of PFS
Months
